# *Paradiplozoon cirrhini* n. sp. (Monogenea, Diplozoidae), a gill parasite of *Cirrhinus molitorella* (Cyprinidae, Labeoninae) in South China[Fn FN1]

**DOI:** 10.1051/parasite/2023022

**Published:** 2023-06-06

**Authors:** Jiayu Huang, Xing Zhou, Kai Yuan, Xuejuan Ding

**Affiliations:** 1 Guangzhou Key Laboratory of Subtropical Biodiversity and Biomonitoring, Guangdong Provincial Key Laboratory for Healthy and Safe Aquaculture, College of Life Science, South China Normal University Guangzhou 510631 China; 2 College of Life Science, Huizhou University Huizhou 516007 China

**Keywords:** Diplozoidae, *Paradiplozoon*, New species, ITS2

## Abstract

*Paradiplozoon cirrhini* n. sp. (Monogenea, Diplozoidae) is described from the gills of mud carp, *Cirrhinus molitorella* (Valenciennes, 1844) (Cyprinidae, Labeoninae), collected in Wuzhou, Guangxi Province, and Conghua, Guangdong Province as part of an ongoing survey of the diplozoid fauna in the Pearl River basin of China. The new *Paradiplozoon* species is distinguished from congeners by the structure of median plate and its outgrowth sclerites. The ITS2 sequences of the new species differ from all known available diplozoid sequences by 22.04%–38.34%. The new species is the first diplozoid species parasitic on Labeoninae in China. Molecular phylogenetic analyses using rRNA ITS2 placed *Paradiplozoon cirrhini* n. sp. in a sister position to the other Chinese *Paradiplozoon*, implying that Labeoninae represents an early and potentially ancestral host group for China *Paradiplozoon*. We also provided ITS2 sequences for four other diplozoids species, namely *P. megalobramae* Khotenovsky, 1982, *P. saurogobionis* (Jiang, *et al.*, 1985) Jiang, Wu & Wang, 1989, *Sindiplozoon hunanensis* Yao & Wang, 1997, and *Sindiplozoon* sp., and validated their phylogenetic position. The results confirm that all diplozoid species are spilt into two major clades and show monophyly of *Sindiplozoon* but paraphyly of *Paradiplozoon*.

## Abstract (fr)

*Paradiplozoon cirrhini* n. sp. (Monogenea, Diplozoidae) est décrit à partir des branchies de la carpe de vase *Cirrhinus molitorella* (Valenciennes, 1844) (Cyprinidae, Labeoninae), collectée à Wuzhou, province du Guangxi, et à Conghua, province du Guangdong dans le cadre d’une enquête en cours sur la faune des Diplozoidae du bassin de la Rivière des Perles en Chine. La nouvelle espèce de *Paradiplozoon* se distingue de ses congénères par la structure de la plaque médiane et ses sclérites d’excroissance. Les séquences ITS2 de la nouvelle espèce diffèrent de toutes les séquences de Diplozoidae disponibles connues de 22,04 % à 38,34 %. La nouvelle espèce est la première espèce de Diplozoidae parasite de Labeoninae en Chine. Les analyses phylogénétiques moléculaires utilisant l’ARNr ITS2 ont placé *Paradiplozoon cirrhini* n. sp. dans une position sœur des autres *Paradiplozoon* chinois, ce qui implique que les Labeoninae représente un groupe d’hôtes précoce et potentiellement ancestral pour les *Paradiplozoon* de Chine. Nous avons également fourni des séquences ITS2 pour quatre autres espèces de Diplozoidae, à savoir *P. megalobramae* Khotenovsky, 1982, *P. saurogobionis* (Jiang, *et al.*, 1985) Jiang, Wu & Wang, 1989, *Sindiplozoon hunanensis* Yao & Wang, 1997 et *Sindiplozoon* sp. et validé leur position phylogénétique. Les résultats confirment que toutes les espèces de Diplozoidae sont réparties en deux clades majeurs et montrent la monophylie de *Sindiplozoon* mais la paraphylie de *Paradiplozoon*.

## Introduction

Monogeneans assigned to the Diplozoidae Palombi, 1949 are blood-feeding ectoparasites that live almost entirely on the gills of freshwater cyprinid fish in Asia, Africa, and Europe [6, 40, 50]. They are a specific group with an unusual life strategy wherein two immature individuals (diporpa) permanently fuse to form an X-shaped sexually mature adult [3]. They fuse their organ tissues such as muscle, nervous, digestive or reproductive, with the vitellaria and most of the intestine in the anterior part of the body, and the reproductive organs and terminal part of the gut in the posterior part of body [56]. The haptor (attachment apparatus) of the adult has four pairs of clamps in two rows and a pair of small central hooks on the ventral side near the first pair of clamps.

Nowadays, the Diplozoidae are divided into two subfamilies: the Diplozoinae Palombi, 1949, and the Neodiplozoinae Khotenovsky, 1980 [25]. The Diplozoinae have five genera (*Diplozoon* von Nordmann, 1832; *Eudiplozoon* Khotenovsky, 1984; *Inustiatus* Khotenovsky, 1978; *Paradiplozoon* Akhmerov, 1974 and *Sindiplozoon* Khotenovsky, 1981), and the Neodiplozoinae have two genera (*Afrodiplozoon* Khotenovsky, 1981 and *Neodiplozoon* Tripathi, 1960) [25]. More than 70 diplozoid species have been reported worldwide, with 37 of them found in China: one species of *Diplozoon*, 26 species of *Paradiplozoon*, two species of *Inustiatus*, one species of *Eudiplozoon*, and seven species of *Sindiplozoon* [2, 5, 8, 13, 49, 50].

Diplozoidae taxonomy is heavily reliant on sclerite construction analysis. These parasites lack sclerotised genitalia and only possess sclerites in their haptor. Their clamps are comprised of a posterior and an anterior jaw that are joined to a median plate by anterior and posterior joining sclerites [25, 39]. These clamps are morphologically distinct and can be used to identify species [7, 44]. However, it has been verified that the measurements of clamps exhibit a relatively high degree of intraspecific variability with different hosts, water temperatures, developmental stage, and geographical origin of parasites [32, 33]. Furthermore, a two-dimensional perspective obtained from conventional light microscopy may result in obscuration of some intricate sclerites that are not perfectly flat or overlapping. Sub-optimal fixing, mounting, and staining protocols add to the difficulties. Therefore, diplozoid species determination based solely on morphological parameters is insufficient.

The importance of combining accurate morphological analysis with molecular analysis is always emphasized as the primary means of identifying diplozoid species [11, 21, 22]. Unfortunately, most of the previous publications lacked representative sequence data, as well as some detailed morphological descriptions. The ITS2 region has been successfully used to distinguish diplozoid species [10, 30, 31, 46], but it seems neither to provide the best resolution for very closely related species complexes nor to be suitable to infer phylogenies for far distantly related species. However, the vast bulk of diplozoid sequence data currently available was generated for the ITS2 fragment.

Most diplozoid species were previously thought to be strictly host specific, with the parasite often pre-determined based on the fish species [19, 25]. However, detailed surveys and molecular analyses revealed that diplozoids have a wider host range than originally believed [6, 15, 46]. Moreover, in case of cyprinids, the correct identification of the fish host is challenged by dimorphisms, geographic variation, as well as hybridisation [43, 51]. To avoid incorrect host identification, molecular data on the host are critical but often overlooked.

The Pearl River is the longest river in south China, with a length of over 2,000 km. It is composed of three separate river systems: the Xi River (originating from the Yunnan-Guizhou Plateau), the Bei River, and the Dong River (both originating from Jiangxi Province). These three rivers meet in Guangzhou, and then flows into the South China Sea. The Pearl River system contains 296 fish species from 17 orders, 45 families, and 156 genera [9]. Cyprinidae account for 146 of these fish species.

Our team has recently investigated the species diversity of monogeneans in the Pearl River system, where the diversity of their potential hosts (cyprinoids) is the highest in China. We collected some representatives of the Diplozoidae specimens, including an undescribed one from the gills of mud carp, *Cirrhinus molitorella*. The host is a freshwater cyprinid native to Asia, with distribution in the Mekong River (Thailand, Laos, and Cambodia), Chao Phraya River (Thailand), Red River (Vietnam), and the Pearl River (China) [36]. This article focuses on the new species’ description and phylogenetic position. Consequently, we provide not only drawings but also photographs of some valued structures of the new species, as well as ITS2 sequences from five species that include this new species.

## Materials and methods

### Sample collection

The host fish, *Cirrhinus molitorella*, were captured in March, April, and June 2015 from the Xijiang River in Wuzhou (111°35′ E, 23°46′ N) of Guangxi Province, and in July and November 2021 from the Liuxihe River in Guangzhou (113°59′–113°71′ E, 23°54′–23°69′ N) of Guangdong Province. The gills were removed from each fish and examined under a microscope for the presence of diplozoids. From 56 host fish, 16 paired adult worms were collected. All worms were gently removed and washed in double-distilled water before being preserved. Nine paired worms were fixed in 70% alcohol for staining, and two paired worms were mounted directly in Berlese’s fluid [55]. The anterior soft parts of five paired worms were preserved in 95% alcohol for DNA extraction, and the posterior haptor parts were separately mounted in Berlese’s fluid or GAP for morphometric analysis [29].

### Morphological methods

Diplozoid parasites preserved in 70% ethanol were stained with acetic carmine, differentiated using HCl in 30% ethanol, dehydrated in graded ethanol series (50%, 70%, 80%, 90%, 95% and 100%), cleared in clove oil, and mounted in Canada balsam [17]. An Olympus BX51 microscope (Olympus, Tokyo, Japan) was used to examine and photograph the specimens. The illustrations were created using an Olympus BX51 microscope’ drawing apparatus and then processed on a computer using Photoshop CS4.0 (Adobe, San Jose, CA, USA). Olympus DP22 software was used to take measurements. Measurements are in micrometres (mm) and are shown as the mean followed by the range and the number of measured specimens in parentheses. The haptoral terminology used herein follows Pečínková *et al.* [38].

### Molecular methods

Total genomic DNA was extracted and purified using a TIANamp Marine Animal DNA Kit (Tiangen Biotech, Beijing, China), as directed by the manufacturer. The ITS2 rDNA was amplified using universal primers of eukaryotes: D (5′–GGC TYR YGG NGT CGA TGA AGA ACG CAG–3′) and B1 (5′–GCC GGA TCC GAA TCC TGG TTA GTT TCT TTT CCT–3′) [4]. Each PCR amplification was carried out in a 50 μL volume containing 25 μL Master Mix (Takara Bio Inc., Kusatsu, Japan), 2 μL genomic DNA (~100 ng), 2 μL of each primer at 10 μM, and 19 μL double-distilled water. Pre-denaturation at 95 °C for 5 min was followed by 35 cycles of 95 °C for 30 s, 55 °C for 30 s, 72 °C for 1 min, and a final extension at 72 °C for 10 min. PCR products were validated by 1% agarose gel electrophoresis and purified using an E.Z.N.A Gel Extraction Kit (Omega Bio-tek, Norcross, GA, USA), according to the manufacturer’s instructions. Purified products were directly sequenced using the PCR primers by the Sangon Biotech Company (Shanghai, China). The sequences were assembled and edited using DNAMAN 7.0 before being compared to the GenBank database content with BLAST.

### Trees and distances

The data obtained for this study and data from GenBank made up the final datasets. Table 1 contains details on these diplozoid sequences. With the aid of several plug-in programs, PhyloSuite was utilised to extract data and perform phylogenetic analysis [54]. Sequences were aligned with MAFFT in PhyloSuite under the G-INS-i iterative refinement algorithm, and removed ambiguously aligned fragments [24]. ModelFinder was used to select the best-fit model using BIC criterion [23]. By using the maximum likelihood method (ML) and Bayesian inference (BI), phylogenetic trees were created with a sequence of *Cemocotyle carangis* Sproston, 1946 as the outgroup. The ML tree was inferred using IQ-TREE under the GTR + G4 + F model for 5000 ultrafast bootstraps [34, 35]. MrBayes 3.2.6 was used to infer the BI tree under GTR + G4 + F model [41], and analyses were performed with 1 million Markov chain Monte Carlo (MCMC) generations for four chains and samples every 100 generations, with the first 25% of trees being eliminated as a relative burn-in period after ensuring that the standard deviation split frequency was less than 0.01. Sequence divergences were estimated in MEGA7.0 using the *p*-distance model [26]. Finally, the trees were embellished on the Itol website after the parasite geographical distributions and host lineages were mapped on to the BI tree and ML tree, respectively [27]. The cyprinoid families are those defined by Tan *et al.* [47].

**Table 1:** List of diplozoid species used in phylogenetic analyses, their fish host species, country of collection and GenBank accession numbers for DNA sequences.

Parasite species	Host species	Fish family, subfamily	Locality	Accession number	Reference
*Diplozoon bliccae* Reichenbach-Klinke, 1961	*Blicca bjoerkna*	Leuciscidae, Leuciscinae	France	AF369761	[46]
*D. kashmirensis* Kaw, 1950	N/A		India	MF460994	
*D. paradoxum* von Nordmann, 1832	*Abramis brama*	Leuciscidae, Leuciscinae	Czech Republic	AJ563372	[30]
*Eudiplozoon kamegaii* Nishihira & Urabe, 2020	*Cyprinus carpio*	Cyprinidae, Cyprininae	Japan	LC517172	[37]
*E. nipponicum* (Goto, 1891) Khotenovsky, 1984	*Cyprinus carpio*	Cyprinidae, Cyprininae	France	AF369758	[46]
*Inustiatus aristichthysi* (Ling, 1973) Jiang, Wu & Wang, 1989	*Hypophthalmichthys nobilis*	Xenocyprididae	China	DQ098894	[15]
*I. inustiatus* (Nagibina, 1965) Khotenovsky, 1978	*Hypophthalmichthys molitrix*	Xenocyprididae	China	DQ098893	[15]
*P. homoion* (Bychowsky & Nagibina, 1959) Khotenovsky, 1985	*Rutilus rutilus*	Leuciscidae, Leuciscinae	China	KP340972	
*P. skrjabini* (Akhmerov, 1974) Khotenovsky, 1985	*Leuciscus baicalensis*	Leuciscidae, Leuciscinae	China	KP340974	
*P. skrjabini* (Akhmerov, 1974) Khotenovsky, 1985	*Phoxinus oxycephalus*	Leuciscidae, Phoxininae	Japan	LC050525	[45]
*P. bliccae* (Reichenbach-Klinke, 1961)	*Blicca bjoerkna*	Leuciscidae, Leuciscinae	Czech Republic	AJ300712	[31]
*P. moroccoensis* Koubková, Benovics & Šimková, 2021	*Luciobarbus lepineyi*	Cyprinidae, Barbinae	Morocco	MT417734	[6]
*P. diplophyllorchidis* (Jiang, *et al.*, 1985) Jiang, Wu & Wang, 1989	*Zacco platypus*	Xenocyprididae	China	DQ098891	[15]
*P. hemiculteri* (Ling, 1973) Khotenovsky, 1985	*Hemiculter leucisculus*	Xenocyprididae	China	KY124645	[21]
*P. helleni* Koubková, Benovics & Šimková, 2021	*Tropidophoxinellus hellenicus*	Leuciscidae, Leuciscinae	Greece	MT417730	[6]
*P. ibericus* Koubková, Benovics & Šimková, 2021	*Squalius valentinus*	Leuciscidae, Leuciscinae	Spain	MT417723	[6]
*P. ichthyoxanthon* Avenant-Oldewage, le Roux, Mashego & van Vuuren, 2013	*Labeobarbus aeneus*	Cyprinidae, Torinae	South Africa	HF566124	[3]
*P. jiangxiense* (Jiang, Wu & Wang, 1985) Jiang, Wu & Wang, 1989	*Chanodichthys erythropterus*	Xenocyprididae	China	DQ098885	[15]
*P. krugerense* Dos Santos & Avenant-Oldewage, 2016	*Labeo rosae*	Cyprinidae, Labeoninae	South Africa	LT574865	[11]
*P. megalobramae* Khotenovsky, 1982	*Megalobrama terminalis*	Xenocyprididae	China	ON907643	Present study
*P. megan* (Bychowsky & Nagibina, 1959) Achmerov, 1974	*Squalius cephalus*	Leuciscidae, Leuciscinae	Czech Republic	AJ300711	[31]
*P. nagibinae* (Gläser, 1965) Khotenovsky, 1985	*Ballerus ballerus*	Leuciscidae, Leuciscinae	Czech Republic	AJ563371	[30]
*P. opsariichthydis* (Jiang, Wu & Wang, 1984) Jiang, Wu & Wang, 1989	*Opsariichthys bidens*	Xenocyprididae	China	MH794188	[22]
*P. parabramisi* (Ling, 1973) Khotenovsky, 1985	*Parabramis pekinensis*	Xenocyprididae	China	DQ098889	[15]
*P. pavlovskii* (Bychowsky & Nagibina, 1959) Khotenovsky, 1985	*Leuciscus aspius*	Leuciscidae, Leuciscinae	Czech Republic	AJ300714	[31]
*P. sapae* (Reichenbach-Klinke, 1961)	*Blicca bjoerkna*	Leuciscidae, Leuciscinae	Czech Republic	AJ300713	[31]
*P. saurogobionis* (Jiang, *et al.*, 1985) Jiang, Wu & Wang, 1989	*Squalidus argentatus*	Gobionidae	China	ON907644	Present study
*P. vaalense* Dos Santos, Jansen van Vuuren & Avenant-Oldewage, 2015	*Labeo umbratus*	Cyprinidae, Labeoninae	South Africa	HG423142	[12]
*P. yarkandense* Arken *et al.*, 2021	*Schizothorax eurystomus*	Cyprinidae, Schizothoracinae	China	MN892630	[2]
*P. yunnanense* Fan *et al.*, 2018	*Sikukia gudgeri*	Cyprinidae, Cyprininae	China	KT781100	[13]
*Sindiplozoon coreius* Cao *et al.*, 2021	*Coreius guichenoti*	Gobionidae	China	MW992745	[8]
*S. ctenopharyngodoni* (Ling, 1973) Jiang, Wu & Wang, 1989	*Ctenopharyngodon idella*	Xenocyprididae	China	DQ098898	[15]
*S. hunanensis* Yao & Wang, 1997	*Squaliobarbus curriculus*	Xenocyprididae	China	ON907645	Present study
*Sindiplozoon* sp.	*Xenocypris davidi*	Xenocyprididae	China	ON907646	Present study
*Paradiplozoon barbi* (Reichenbach-Klinke, 1951) Khotenovsky, 1985	N/A		N/A	MN688771	
*P. bingolensis* Civáňová, Koyun & Koubková, 2013	*Garra rufa*	Cyprinidae, Labeoninae	Turkey	HE653910	[10]
*Paradiplozoon cirrhini* n. sp.	*Cirrhinus molitorella*	Cyprinidae, Labeoninae	China	ON907642	Present study
				OQ429337	Present study
				OQ429338	Present study

## *Paradiplozoon cirrhini* n. sp. (Figs. 1–4)

urn:lsid:zoobank.org:act:DAC5F1C0-3955-4D5E-AF2B-7C5C478B0EB0

**Figure 1:**
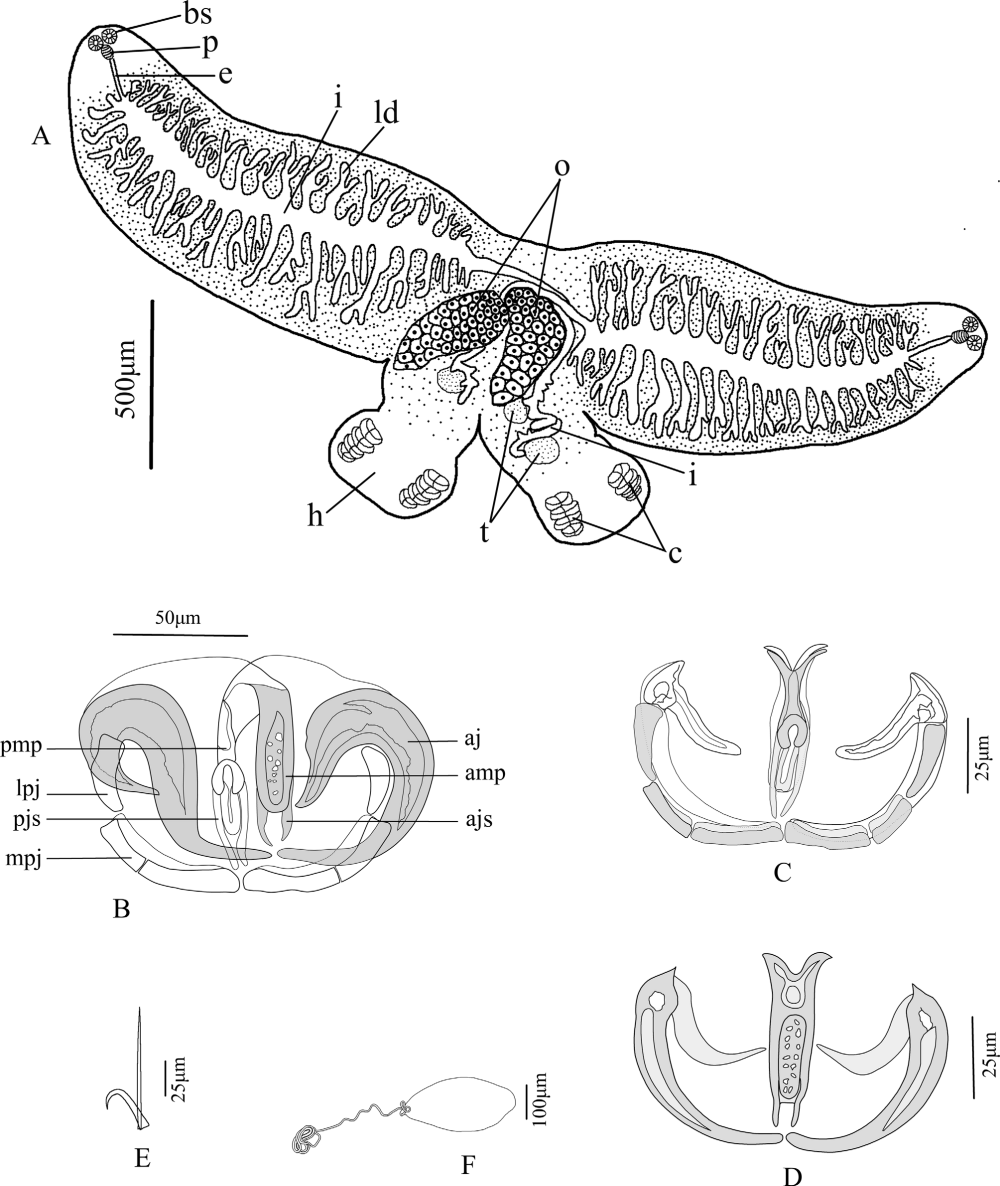
Line drawings of *Paradiplozoon cirrhini* n. sp. A. Whole worm, ventral view (bs, buccal sucker; p, pharynx; e, esophagus; i, intestine; ld, lateral diverticula; o, ovary; t, testis; h, haptor; c, clamp); B. Clamp in somewhat lateral view (amp, anterior end of the median plate; pmp, posterior end of the median plate; mpj, medial sclerite of posterior jaw; lpj, lateral sclerite of posterior jaw; aj, anterior jaw; ajs, anterior joining sclerites; pjs, posterior joining sclerite); C. Clamp in posterior view; D. Clamp in anterior view; E. Central hook; F. Egg.

Type-host: *Cirrhinus molitorella* (Valenciennes, 1844).

Site of infection: Gills.

Type locality: Xijiang River in Wuzhou (111°35′ E, 23°46′ N) of Guangxi Province in China.

Type material: Holotype (GXWZ. 2015033101), 20 paratypes (GXWZ. 2015033102-09, 2015040101-02, 2015060401, GDGZ. 2021070901-05, 2021110101-04), deposited in the Laboratory of Fish Parasite, College of Life Science, South China Normal University, Guangzhou, China. One paratype (MNHN HEL1928), deposited in the Collections of the Muséum National d’Histoire Naturelle, Paris (MNHN).

Etymology: The new species is named after its host.

### Description

Two adult individuals display typical X-shape body, divided into fore- and hindbody, with total body length of 2.813 (1.376–6.314, *n* = 9), tegument smooth. Forebody 1.920 (0.866–4.434, *n* = 9) long and 0.668 (0.443–1.107, *n* = 9) wide. Hindbody 0.757 (0.442–1.289, *n* = 9) long from the fusion area to the end of haptor, no “cup-like” widened area (Figs. 1A, 3A). One pair of buccal suckers elliptical, opening sub-terminal, 0.071 (0.041–0.110, *n* = 9) × 0.078 (0.044–0.115, *n* = 9), glandular structures absent (Fig. 1A). Pharynx ovate, immediately near buccal suckers, 0.052 (0.038–0.064, *n* = 9) × 0.070 (0.058–0.094, *n* = 9), opening into highly branched intestine (Fig. 1A). Intestine extending through region of reproductive organs and ending near haptor, with prominent lateral diverticula in forebody (Fig. 1A). From posterior to pharynx, to onset of fusion region, anterior region displays large number of vitellaria (Fig. 3B). Reproductive organs located in anterior part of hindbody (Figs. 1A, 3A). Ovary kidney-shape with one elongated projection to link backward curved oviduct (Fig. 3B). Testes two, arranged in front and back (usually front one covered by ovary or intestine), solid and oval-shaped, posterior to ovary (Figs. 1A, 3B). Egg 0.333 (0.327–0.338, *n* = 2) × 0.151 (0.133–0.170, *n* = 2) in size, with filament on one end, filament length 1.068 (0.965–1.24, *n* = 2) (Figs. 1F, 3E).

Haptor disc-like, 0.268 (0.202–0.344, *n* = 10) × 0.428 (0.308–0.657, *n* = 10), with four pairs of clamps and one pair of central hooks in each haptor. Clamps of adult worms smaller in size towards posterior end of haptor (Figs. 4a–4e), first clamp (most posterior) 0.060 (0.031–0.105, *n* = 14) × 0.96 (0.61–0.142, *n* = 14), second clamp 0.065 (0.033–0.104, *n* = 15) × 0.120 (0.074–0.187, *n* = 15), third clamp 0.067 (0.036–0.112, *n* = 15) × 0.127 (0.083–0.215, *n* = 15), the fourth clamp 0.073 (0.041–0.119, *n* = 15) × 0.126 (0.081–0.195, *n* = 15). Central hook sickle 0.014 (0.011–0.018, *n* = 6) long, hook handle 0.036 (0.026–0.041, *n* = 6) long (Figs. 1E, 3D).

**Figure 2:**
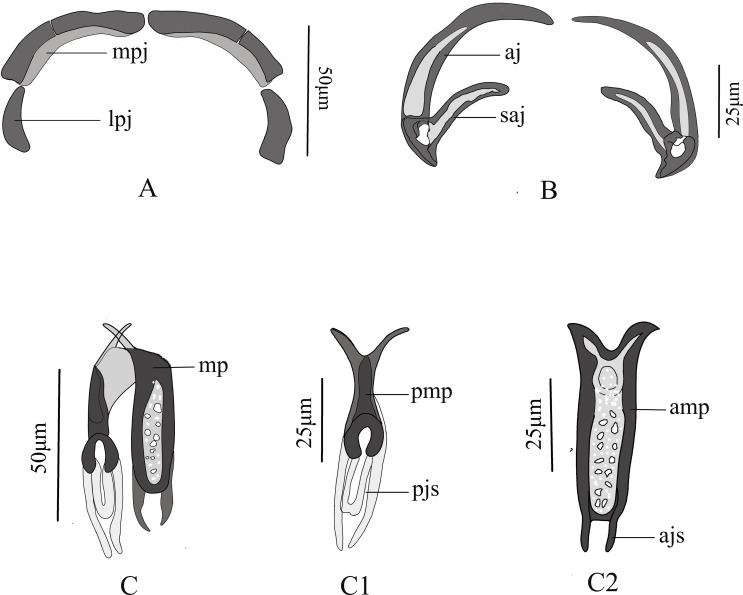
Line drawings of clamp sclerites of *Paradiplozoon cirrhini* n. sp. A. Medial sclerite of posterior jaw (mpj) and lateral sclerite of posterior jaw (lpj); B. Anterior jaw (aj) with a spur of the anterior jaw (saj); C. Median plate (C1, median plate in posterior view; C2, median plate in anterior view).

**Figure 3:**
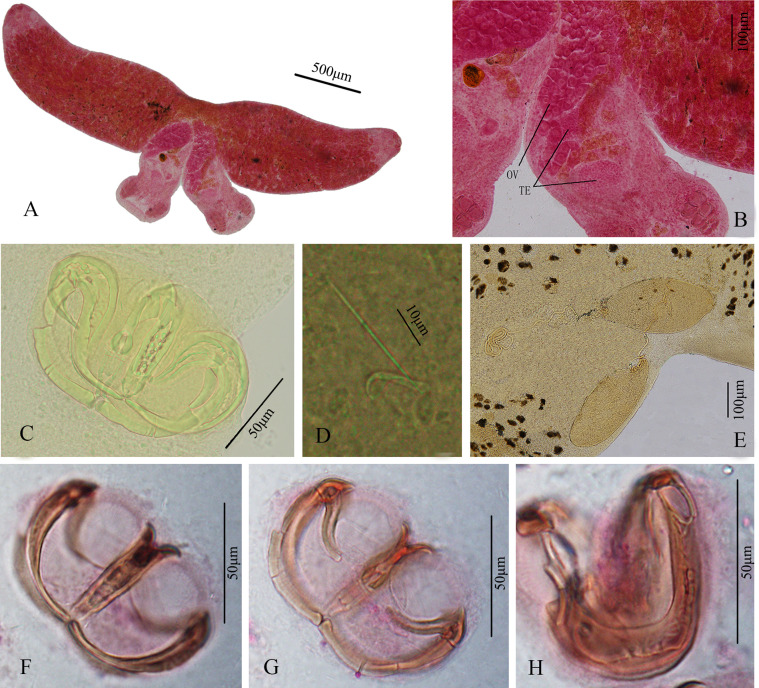
Photographs of *Paradiplozoon cirrhini* n. sp. A. Holotype, whole worm (ventral view); B. Reproductive system (te, testis; ov, ovary); C. Clamp in somewhat lateral view; D. Central hook; E. Eggs; F. Clamp sclerites in front view; G. Clamp sclerites in back view; H. Median plate in lateral view.

Each clamp consists of sclerotised structures: median plate(mp), anterior joining sclerite (ajs), posterior joining sclerite (pjs), anterior jaw (aj), medial sclerite of posterior jaw (mpj), and lateral sclerite of posterior jaw (lpj) (Figs. 1B, 2). Different clamps components can be seen from different viewing angles or focal planes (Fig. 1C *vs* 1D, Fig. 3F *vs* 3G). Median plate u-shaped in lateral view (Figs. 2C, 3H), with the bottom expanding outwardly to form a Y-shaped in frontal view (Figs. 2C1–2C2). Anterior end of median plate (amp) rectangular with thick edges and many perforations in central area (Figs. 2C2, 3C). Posterior end of median plate (pmp) narrows and terminates with wide-rounded sclerite with opening (Fig. 2C1). Trapeze spur absent. Two thin anterior joining sclerites (ajs) poorly visible, protruding parallel from edge of median plate to proximal tip of anterior jaw (Figs. 2C2, 3H). Posterior joining sclerite (pjs) consists of u-shaped medial and two slender lateral sclerites with their ends close (Figs. 2C1, 3C). Anterior jaws (aj) typical in shape of big hook, with obvious circular perforation at junction with spur of anterior jaw (saj) (Fig. 2B). The medial sclerite of posterior jaw (mpj) wide and flaky with a weakly visible suture approximately in middle (Fig. 2A).

### Differential diagnosis

*Paradiplozoon* is the most speciose genus of the Diplozoinae, and its clamp structure, particularly the median plate and its outgrowth structure (trapeze spur, anterior or posterior joining sclerites), is considered the most important morphological character for species discrimination [12, 20, 25, 30, 31]. In comparison to related species, this new species differs in the following characteristics. The anterior end of the median plate has numerous continuous perforations that extend almost the entire length of median region. The anterior joining sclerites are small and project parallel from the outer edge of the median plate (the trapeze spur is absent). The posterior joining sclerites consist of two slender lateral sclerites with closely spaced ends, and a u-shaped medial sclerite. The comparison of morphometrics of related *Paradiplozoon* spp. is shown in Table 2. Although the measurements of the central hook are normally considered to be taxonomically significant, the central hook of this new species overlaps with those of other species.

**Table 2:** Measurements of *Paradiplozoon cirrhini* n. sp., and related *Paradiplozoon* spp.

	*P. cirrhini* n. sp.	*P. saurogobionis* (Jiang *et al.*, 1985) Jiang, Wu et Wang, 1989	*P. megalobramae* Khotenovsky, 1982	*P. yunnanensis* Fan *et al.*, 2018	*P. hemiculteri* (Ling, 1973) Khotenovsky, 1985	*P. opsariichthydis* (Jiang, Wu & Wang, 1984) Jiang, Wu & Wang, 1989	*P. yarkandense* Arken et al, 2021
Hosts	*C. molitorella*	*S. argentatus*	*M. terminalis*	*S. gudgeri*	*H. leucisculus*	*O. bidens*	*S. eurystomus*
Source	present study	present study	present study	[13]	[21]	[22]	[2]
Body length	2813 (1376–6314)	1762–3654	6175–7375	1593 (1148–2344)	3153 (2320–4180)	3840 (2926–4457)	2130 (1100–3050)
Body width	668 (443–1107)	592–997	962–1225	445 (211–626)	931 (729–1140)	991 (739–991)	690 (440–1010)
Haptor length	268 (202–344)	168–698	300–450	–	–	–	340 (240–510)
Haptor width	428 (308–657)	308–548	513–725	–	–	–	300 (180–410)
Oral sucker	71 (41–110) × 78 (44–115)	62–98 × 62–88	85–90 × 90–100	46 (23–65) × 35 (26–44)	58 (50–67) × 54 (42–65)	66 (52–78) × 60 (47–62)	60 (50–80) × 50 (40–70)
Pharynx	52 (38–64) × 70 (58–94)	38–85 × 41–73	60–70 × 50–70	45 (30–56) × 38 (21–52)	66 (50–83) × 54 (38–62)	60 (55–68) × 50 (44–57)	47 (46–49) × 42 (41–43)
1st pair clamps	60 (31–105) × 96 (61–142)	98–134 × 138–211	87–115 × 115–150	39 (18–59) × 53 (23–78)	56 (42–70) × 90 (72–119)	80 (57–79) × 140 (125–179)	70 (50–80) × 110 (90–120)
2nd pair clamps	65 (33–104) × 120 (74–187)	104–169 × 151–264	77–140 × 130–192	44 (20–65) × 61 (27–86)	60 (49–69) × 104 (91–115)	89 (86–137) × 164 (160–198)	90 (80–100) × 150 (140–180)
3rd pair clamps	67 (36–112) × 127 (83–215)	107–153 × 166–274	107–142 × 155–205	43 (17–61) × 62 (29–89)	61 (47–82) × 107 (74–127)	92 (80–138) × 172 (161–207)	100 (90–110) × 160 (140–190)
4th pair clamps	73 (41–119) × 126 (81–195)	101–157 × 161–253	100–140 × 115–187	46 (20–59) × 62 (27–87)	62 (47–82) × 105 (76–126)	91 (77–143) × 172 (161–207)	80 (70–90) × 140 (120–170)
Hook sickle	14 (11–18)	15–18	15–17	12	17.6 (17–18.1)	15.1 (12.5–16.5)	23 (19–26)
Hook handle	36 (26–41)	37–41	32–42	21.5	36.6 (34–37.9)	36.6 (31.9–38.4)	42 (39–45)
Egg	333 (327–338) × 151 (133–170)	–	200–210 × 90–100	151 (142–160) × 75 (74–87)	–	–	200 (180–220) × 70 (60–80)

### Molecular analyses

DNA sequences amplified from the ITS2 fragment of three adult worms were generated, and deposited in GenBank under accession numbers ON907642 (806 bp), OQ429337 (809 bp), and OQ429338 (813 bp). All three sequences are highly similar, with only several base variations at opposite ends of the sequence. The BLAST result indicated less than 80% identity with 98%–99% coverage for other *Paradiplozoon* monogeneans in GenBank, with the highest similarity (78.41%) to *P. hemiculteri* (DQ098892) and *P. diplophyllorchidis* (DQ098891). Here we also provide ITS2 sequences for four additional diplozoids species, namely *P. megalobramae* (ON907643), *P. saurogobionis* (ON907644), *S. hunanensis* (ON907645) and *Sindiplozoon* sp. (ON907646).

Representative sequences of 35 other diplozoids were selected from either different geographical regions or phylogenetically divergent host species. After aligning the data, the final dataset contained 656 positions, including 168 bp conserved sites, 488 bp variable sites, and 417 bp parsimony-information sites. The genetic distances between *P. cirrhini* n. sp. and other members of Diplozoidae ranged from 22.04% to 38.34% (Supplementary Table 1). The most closely related species to *P. cirrhini* n. sp. were *P. megalobramae* (ON907643), *P. hemiculteri* (KY124645), and *P. opsariichthydis* (MH794188), with estimated genetic distances of 22.04%, 22.61% and 23.23%, respectively.

For trees, the Bayesian inference (Fig. 5) and maximum likelihood (Fig. 6) methods led to identical topologies. Both trees show that all diplozoid taxa are split into two major evolutionary lineages, and further divided into five clades. The first lineage consists of two well-supported sister clades: clade 1 is composed of a single species of *Afrodiplozoon* from Africa and four species of *Paradiplozoon* from Africa, Turkey and China, and clade 2 is made up of nine species of *Paradiplozoon* from China. The second lineage is represented by species of *Paradiplozoon* and *Diplozoon*, most of which come from Europe, as well as members of *Inustiatus*, *Eudiplozoon*, and *Sindiplozoon*. In both ML and BI analyses, *Inustiatus* and *Eudiplozoon* formed a basal group (clade 3) to all other diplozoids in second lineage, but the nodes for this were not well supported in ML tree. *Sindiplozoon* taxa (clade 4) is consistently sister to European *Paradiplozoon* and *Diplozoon* taxa (clade 5) across all methods used, with fairly high statistical support.

The three sequences of newly described species (*P. cirrhini* n. sp.) formed a well-supported monophyletic group, and then clustered with other China *Paradiplozoon* spp., all of which form a sister group (clade 2) to species from South Africa (*P. krugerense* LT574865, *A. polycotyleus* LT719088), northwest African (*P. moroccoensis* MT417734), Turkey (*P. bingolensis* HE653910), and west of China (*P. yarkandense* MN892630) in clade 1.

## Discussion

**Figure 4:**
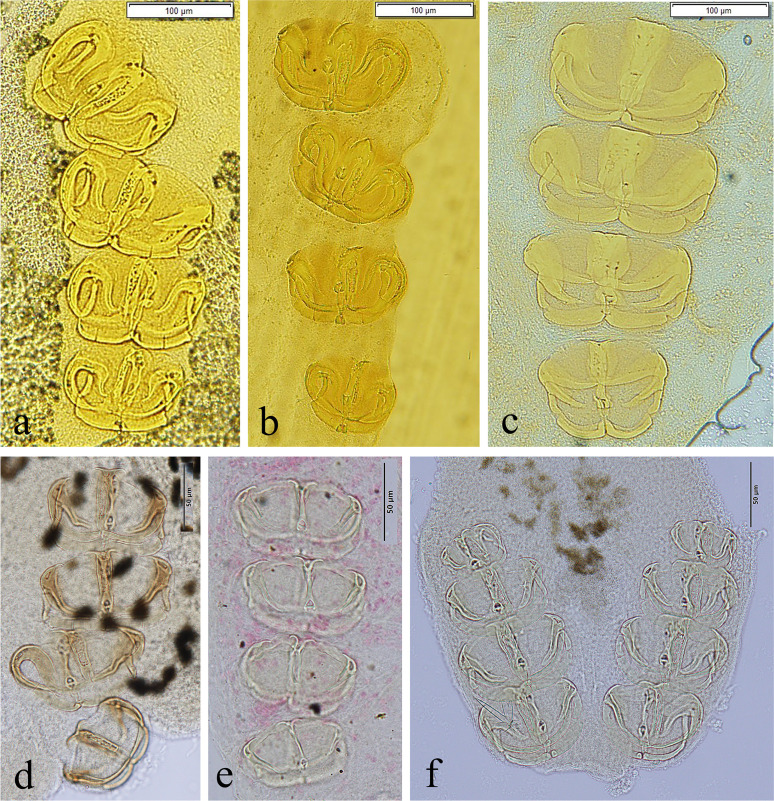
Photographs of clamps in different specimens of *Paradiplozoon cirrhini* n. sp. a–e are from adult worms, f is from a diporpa.

The Pearl River is located in a subtropical karst region and has a rich and distinctive freshwater fish community [9]. It exhibits a faunal succession from north to south in Eastern Asian [28]. However, the diversity of monogeneans in this system has been underestimated. All data presented in this article are part of ongoing research to document the diplozoid fauna in the Pearl River system. Our study presents the results of a detailed morphological and morphometric description of *P. cirrhini* n. sp., combined with molecular identification using ITS2 as a genetic marker. Their sclerotised structures were studied exclusively by light microscopy of mounted specimens. Even though the new species is easily distinguished from others, morphological variations in the clamps were also observed (Fig. 4). Clamps of diplozoids are typically structurally complex. Fixation and preparation, as well as the degree of pressure applied to the coverslip during fixation and mounting, can all result in skewed measurements and observations. As a result, some previous drawings of key morphological features were neither consistent nor always accurate [21, 22]. The high genetic difference (22.04% to 38.34%) from all other diplozoids further confirmed the uniqueness of the current species. Like the previous analyses [15, 22], our analysis revealed very low genetic difference (0.00%–0.31%) between the following species: *P. jiangxiensis*, *P. opsariichthydis*, *P. parabramisi*, and *P. diplophyllorchidis*. Jirsová *et al.* [22] undertook a redescription of *P. opsariichthydis* and considered that these species should be identified with *P. opsariichthydis* or referred to as the *P. parabramisi*-complex, as suggested by Dos Santos & Avenant-Oldewage [11], before additional markers (such as COI) alongside expanded morphometric analyses helped to clarify this issue. It has been amply demonstrated that the use of just one of both approaches is insufficient and can lead to controversial conclusions [5, 15]. Of 38 species recorded in China, only nine species are validated by both morphological and molecular data ([2, 8, 13, 21, 22], present study). Morphological re-evaluation of diplozoid species of China in combination with DNA sequencing is urgently needed.

Our phylogenetic reconstructions revealed that all diplozoid species are split into two major lineages as shown previously [2, 6, 11]. The monophyly of *Inustiatus*, *Eudiplozoon*, and *Sindiplozoon* was strongly supported by the high support value of their own branches. All recent phylogenetic studies ([2,15], present study) on diplozoid parasites confirmed the paraphyly of the *Paradiplozoon*, making the revised proposal of “Genus 1–3” by Dos Santos & Avenant-Oldewage for *Paradiplozoon* reasonable [11]. However, there are currently no robust morphological criteria for distinguishing these putative taxa, especially because *Afrodiplozoon* (Neodiplozoinae) nests within the *Paradiplozoon* group. We noted that our trees based on ITS2 data have low root support nodes. Although our analyses place *Inustiatus* and *Eudiplozoon* in the same clade, the contradiction with other studies means that the placement of these genera requires further confirmation [10]. In contrast to previous studies on the ITS-2 sequences, *Eudiplozoon* formed a sister group to all other available Diplozoidae in more recent studies on mitogenome data [18, 53]. Although mitogenome analyses did not cover representatives of all groups, they provided us with a plausible scenario about the phylogenetic origin of diplozoid species.

**Figure 5:**
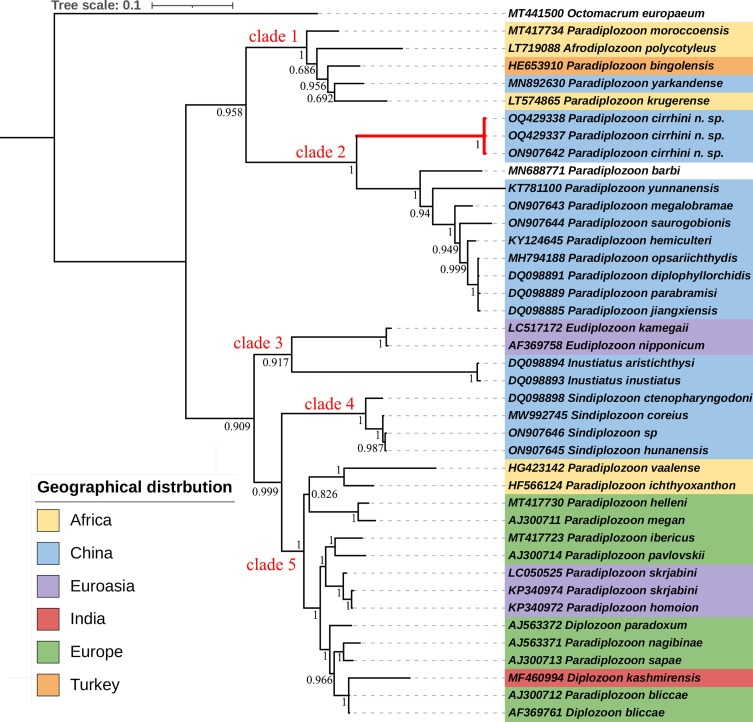
Mapping of the parasite geographical distribution onto the BI tree inferred from analyses of ITS2 sequences of selected diplozoids. The numbers at nodes indicate posterior probabilities (%); *Paradiplozoon cirrhini* n. sp are highlighted by red branches. Clades 1–5 represent the well-supported groups described in the “Results”.

Diplozoids have a wide distribution in Eurasia and Africa. To reveal their phylogeographic origin, we mapped the geographic distribution on the BI tree (Fig. 5). From the topology, we can see some obvious phylogeographic patterns. The first lineage consists of species from Asia (China), Africa, and the Middle East. Clade 1 consists of species from South Africa (*P. krugerense*, *A. polycotyleus*), northwest African (*P. moroccoensis*), Turkey (*P. bingolensis*), and west of China (*P. yarkandense*). *Paradiplozoon yarkandense* has been reported only from the Yarkand River (a tributary of the Tarim River) in Xinjiang Province, which is located in the westernmost part of China and has more natural geographic connections to Central Asia [2]. *Paradiplozoon bingolensis* from Turkey is thought to be a link between Europe, Asia and Africa [10, 40]. Owing to their phylogenetic proximity, Benovics *et al.* [6] assumed that species of this clade have a common origin in the Middle East/Asia. All *Paradiplozoon* specimens collected in China are assigned to clade 2, including one species of *P. barbi* recoded only from Malaysia. Interestingly, species from river systems in southern China (*P. cirrhini* n. sp. and *P. megalobramae* from the Perl River, *P. yunnanensis* from the Lancang-Mekong River) were placed at the basal position. The results indicate that China *Paradiplozoon* most likely originated in the ancient Pearl River basin and then migrated northward into the Yangtze River basin and southward into Southeast Asia. Given the wide distribution of *Cirrhinus molitorella* [36], *P. cirrhini* n. sp. could possibly exist in Southeast Asia. Therefore, we cannot rule out the possibility that they originated in Southeast Asia and then spread northward across the Pearl River into northern China. Our phylogenetic analyses support Benovics’ assumption that the first lineage species originated in Asia, most likely Southeast Asia, and spread to Africa *via* the Middle East [6].

The second lineage consists of species from Europe, Asia, and Africa. Both *Inustiatus* and *Sindiplozoon* occur exclusively in China, and *Eudiplozoon* sensu stricto is distributed only in East Asia [37, 50]. These three genera were placed at the root of the second lineage, suggesting that this lineage is of East Asian origin and diversifies primarily in Europe. All *Paradiplozoon* specimens collected in Europe are included in Clade 5 alongside all *Diplozoon* species. It is noteworthy that three Chinese *Paradiplozoon* sequences (*P. homoion* KP340972, *P. skrjabini* KP340974 and *P. gracile* KP340973) [11], two African sequences (*P. vaalense* HG423142 and *P. ichthyoxanthon* HF566124) [12, 40], and one Indian sequence (*D. kashmirensis* MF460994) [1] are all nested within the European group. Although the three species, *P. homoion*, *P. skrjabini* and *P. gracile*, have been previously reported from other localities in Eurasia, their sequences used in our study were most likely collected in Xinjiang Province according to the authors. *Diplozoon kashmirensi*s has been collected in India’s Kashmir Valley, as well as in Kazakhstan [1]. These findings suggest that European taxa may have multiple origins, with the Middle East possibly serving an intermediary.

**Figure 6:**
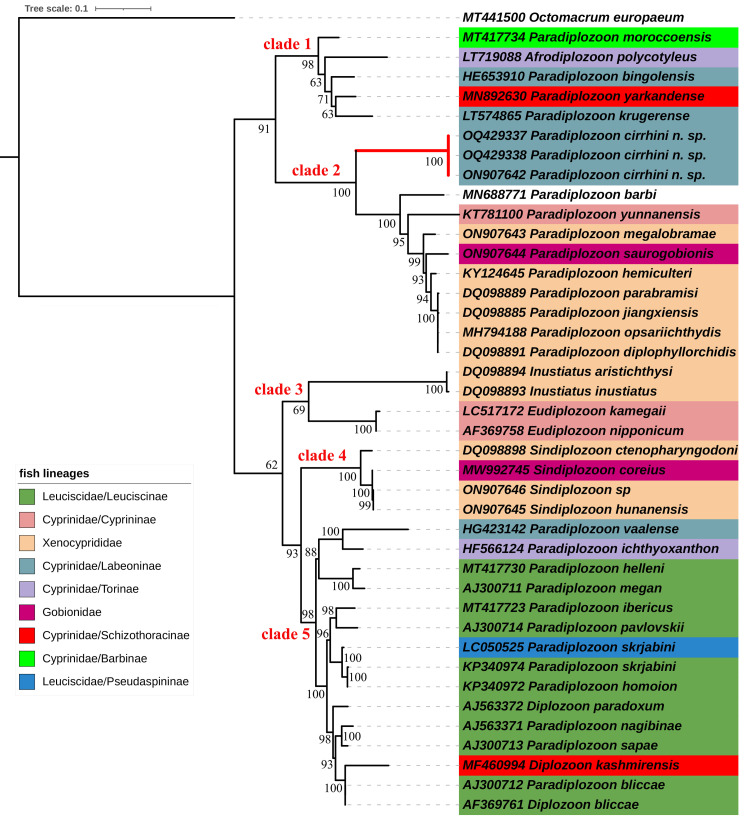
Mapping of fish lineages onto the ML tree inferred from analyses of ITS2 sequences of selected diplozoids. The numbers at nodes indicate bootstrap values (%); *Paradiplozoon cirrhini* n. sp are highlighted by red branches.

The phylogeographic origin of parasites and their historical dispersion are intimately linked with the phylogeography of their hosts. Coevolution is not the focus of this study, but we briefly discuss several interesting points about biogeographical origin from the host perspective. Both paleontological evidence and molecular phylogenetic reconstructions suggest that the cyprinoids originated from the Oriental subtropics [16, 48]. This is one of the reasons why the Asian origin of diplozoids taxon is prioritised. We herein mapped the host lineages onto the ML tree (Fig. 6). As can be seen, Chinese diplozoids primarily parasitise the Xenocypridinae fish. The Xenocyprinae are a highly diverse cyprinoid taxon that arose in the “Yangtze River-Pearl River” basin after the Tibetan Plateau uplift from 25 to 20 Mya, and thrived intimately with subsequent monsoon-driven climatic conditions in East Asia (especially in China) [14]. Such a rapid radiation of xenoprinines is potentially followed by the cospeciation of their *Paradiplozoon* parasites in geographically isolated regions. The new species is the only Chinese representative from Labeoninae (seems strictly host-specific to the mud carp *Cirrhinus molitorella*). The host mapping revealed that Labeoninae are an evolutionary old host group for China *Paradiplozoon*. Within clade 1, Diplozoids of Labeoninae are also present in Middle East (*P. bingolensis*) and Africa (*P. krugerense*). We could hypothesise that the historical origin of species in clade 1 is associated with the historical Oriental-to-Afrotropical migration of labeonines *via* connection of the African and Arabian or Indian plates [52]. The European diplozoids exclusively parasitise the Leuciscidae in our analyses. Different Leuciscidae clades are found in Eurasia and North America [42]. Due to a lack of representatives of Leuciscidae native to other locations, we are unable to determine specific origins of their diplozoid. However, *Sindiplozoon*’ association with European diplozoids implies that Xenocypridinae or Gobionidae seem to be an early and potentially ancestral host group for European diplozoids, and Leuciscidae represent a more recently evolved host group. The fact that European diplozoids are nested with species from Cyprininae, Barbinae, Labeoninae, and Schizothoracinae of Cyprinidae suggests that they have a complex evolutionary scenario ([1, 11], present study).

Nonetheless, it is important to note that all these above phylogenetic analyses are based on only ITS2 for a limited number of diplozoid species. This marker has its own discriminatory power limitations for inferring phylogenies [11]. Comprehensive multilocus studies are needed for diplozoid taxonomy. Records of China diplozoids infecting other fish subfamilies do exist, such as Torinae, Cyprininae, Gobioninae, Opsariichthyinae, Leuciscinae, and even Channidae, Acheilognathidae, Cobitidae, and Botiidae [53]. Thus, an extensive investigation of hidden diplozoid diversity in China (however, in other regions, such as Southeast Asia, Middle Asia, as well) and studies focused on the coevolution between cyprinoids and diplozoids may shed light on the origin and historical dispersion of this group.

## Supplementary material

The supplementary material of this article is available at https://www.parasite-journal.org/10.1051/parasite/2023022/olm. *Supplementary Table 1*: Genetic distances between *P. cirrhini* n. sp. and other members of the Diplozoidae.
